# Impact of Drug-to-Drug Interactions on Patients With Advanced Cancer in Palliative Care Settings

**DOI:** 10.7759/cureus.106324

**Published:** 2026-04-02

**Authors:** Hassan M Albadour, Dalal S Salem, Eiman A Hussain, Hiba Osman, Lamia A Basiouny, Hala Srrani, Ohoud S AlAmri, Mohamed A Alshaqi

**Affiliations:** 1 Palliative Care, Armed Forces Hospital Southern Region, Khamis Mushayt, SAU; 2 Oncology, Armed Forces Hospital Southern Region, Khamis Mushayt, SAU

**Keywords:** adverse drug reactions, drug-to-drug interactions, oncology and end of life, palliative care, polypharmacy

## Abstract

Background: Drug-to-drug interactions (DDIs) pose a significant challenge in managing elderly patients with chronic illnesses and polypharmacy. Similarly, patients with advanced malignancies often experience complex symptoms that necessitate the use of medications such as narcotics, tranquilizers, laxatives, antiemetics, and antipsychotics, drug classes known for their high risk of severe and potentially fatal interactions.

Aim: The aim of this study is to assess the prevalence of DDIs, their categories and clinically significant adverse events among palliative care patients in Armed Forces Hospital, Southern Region, Saudi Arabia.

Methods: We conducted a prospective cross-sectional study involving palliative patients with advanced malignancies at the Palliative Medicine Department of Armed Forces Hospital, Southern Region, Saudi Arabia. The study evaluated the number of prescribed medications, the occurrence and categories of DDIs, their clinical significance, and the strategies used for management.

Results: We enrolled 40 patients with advanced malignancies, with a mean age of 74 years (±14.5), ranging from 27 to 93 years, and nearly equal distribution of male patients (52.5%) and female patients (47.5%). DDIs were reported in 75% of the patients, with the majority (66.1%) classified as moderate in severity. Only 17.5% of patients experienced adverse effects related to DDIs, with sedation and confusion being the most frequently reported. Patients with DDIs also tended to have more severe symptoms, particularly pain, depression, and appetite loss. Polypharmacy, with a mean of 10.3 medications per patient, emerged as a key factor contributing to DDIs.

Conclusion: DDIs are relatively common among palliative care patients, but clinically significant adverse events are infrequent. Given the small sample size and single-center design, findings should be interpreted with caution. Regular medication reviews and tailored prescribing may support safer, more patient-centered care at the end of life.

## Introduction

Palliative care is a specialized field of medicine focused on managing patients with incurable diseases and limited life expectancy [1]. The World Health Organization (WHO) has estimated that approximately 40% of patients diagnosed with advanced malignancies and who need palliative care services are aged ≥70 years [2].

Studies have shown that 86% of elderly patients receiving palliative care have multiple comorbidities (43% with ≥4 chronic conditions) alongside advanced malignancies or other progressive diseases that classify them as palliative [3].

Globally, polypharmacy, defined as a patient taking five or more concurrent medications, is observed in 37% of the general population and 59% in elders, with the highest rates reported in Europe, accounting for 68% [4]. It exceeds 90% in palliative care due to the complexity of symptom management [5]. Drug-to-drug interactions (DDIs) are frequently observed in palliative care, with retrospective studies reporting prevalence rates between 31% and 63%, with moderate severity being the most common category [6-10].

The most frequently prescribed medications in palliative care include narcotics, tranquilizers, laxatives, antiemetics, and antipsychotics. In contrast, antihypertensives, hypoglycemics, anti-heart failure medications, and non-steroidal anti-inflammatory drugs are commonly used among elderly patients [11]. These medications pose significant risks of DDIs, potentially leading to sedation, central nervous system depression, delirium, extrapyramidal side effects, arrhythmias, and QT prolongation [12,13].

Despite the growing integration of artificial intelligence and digital transformation in healthcare, electronic medical record systems are now equipped with automated DDI detection using computerized clinical decision support systems (CDSS) [14,15]. However, in palliative care, where symptom relief often requires a subtle approach, physicians frequently need to override CDSS alerts to prescribe essential medications despite known DDIs.

A systematic review and meta-analysis revealed that the CDSS generated alerts at an overall prevalence of 13%, physicians overrode these alerts at a high rate of 90%, these findings suggest that optimizing CDSS alerts, particularly in the context of palliative care, should consider the limited applicability of DDI alerts for patients whose primary goal of care is symptom relief and quality of life [16].

There is a lack of evidence supporting this practice, and further research is needed to validate the clinical judgment of palliative care physicians in balancing symptom management with medication safety [6,7,9,10].

Given the limited number of studies, most of which are retrospective, assessing the prevalence and outcomes of DDIs in palliative care, factors such as short life expectancy and a focus on symptom palliation may contribute to the underreporting of DDIs in this population [6,7,9,10]. Therefore, we conducted a prospective cross-sectional study to evaluate the prevalence, categories, and clinical outcomes of DDIs among palliative care patients with advanced malignancies.

## Materials and methods

We conducted a prospective cross-sectional study of patients diagnosed with advanced malignancies who were not candidates for or actively receiving treatment in the Palliative Medicine Department of Armed Forces Hospital Southern Region, Saudi Arabia.

A total of 40 patients were enrolled between December 2, 2024, and April 1, 2025. Eligible participants were those admitted to inpatient wards with advanced malignancies who were no longer candidates for active oncological treatment. Patients receiving active cancer therapies (chemotherapy, definitive radiotherapy, or hormonal treatment) or those who withdrew consent at any point during the study were excluded.

All prescribed drugs were entered into Micromedex drug-drug interactions checker and revalidated using the Medscape drug-to-drug interaction checker [17]. 

After obtaining written informed consent, we collect data regarding demographics such as age, gender, diagnosis, and date of admission, as well as chronic illnesses such as hypertension, diabetes, chronic kidney disease, ischemic heart disease, Cerebrovascular stroke, and chronic liver disease, along with symptoms and their severity, using ESAS-R (Edmonton Symptom Assessment System-Revised) which is a validated, patient-reported instrument designed to provide a rapid and clinically relevant assessment of nine common symptoms in palliative care patients. It included 10 cardinal symptoms commonly reported at the end of life, the severity of symptoms is presented on a 0-10 scale, and was collected longitudinally over multiple time points, initially at admission and every 48 hours during admission duration [18].

Regarding drugs, we collected the total number of medications per patient, the number of potential drug interactions per patient using Micromedex and Medscape drug-to-drug interaction checker, and the number of minor DDIs defined as mild and do not significantly impact the patient outcome, as well as the number of intermediate DDIs defined as clinically significant that require careful consideration by the healthcare provider, the number of major DDIs defined as potentially life-threatening or can cause fatal outcomes or prolonged hospitalization, and the number of patients who developed clinically significant DDIs, their management, and ultimate clinical outcome.

The study objectives were to assess the prevalence of DDIs among palliative care patients in the Armed Forces Hospital, Southern Region, Saudi Arabia. As secondary objectives, the study evaluated the prevalence of types of DDIs and determined how frequently these interactions lead to clinically significant outcomes.

DDIs were categorized into potential and actual DDIs, as potential DDIs are a theoretical possibility that one drug may alter the intensity of the pharmacological effect of another drug given concurrently, as extracted from software, while actual DDIs are defined as manifested as clinical symptoms, measured as laboratory abnormality, or an adverse drug reaction (ADR) in the patient.

To ensure objective attribution and minimize bias, causality was determined using the Naranjo Adverse Drug Reaction Probability Scale. Furthermore, independent adjudication was performed by two senior palliative care consultants; in cases of disagreement, a third senior clinician (Biostatistician/Clinical Pharmacologist) served as the tie-breaker to reach a final consensus.

Ethical consideration

The study was conducted in accordance with the ethical principles outlined in the Declaration of Helsinki. Prior to enrollment, all participants provided written informed consent after receiving a full explanation of the study’s objectives and their right to withdraw at any time without penalty. To ensure participant privacy, all data were anonymized and stored on secure, password-protected systems. The study protocol and informed consent were submitted to the AFHSR institutional review board, and approval was granted on December 1st, 2024, with code (AFHSRMREC/2024/palliative care unit/ 746).

The sample size was determined using MedCalc® Statistical Software version 20.215 (MedCalc Software Ltd, Ostend, Belgium). Based on a literature review, the reported prevalence of DDIs among palliative care patients ranged from 33% to 75% [6-10]. To account for this variability, we assumed an expected DDI prevalence of 50%, with a precision of ±30%, allowing us to detect a prevalence range from 20% to 80% within a 95% confidence interval. This yielded a minimum required sample size of 39 patients. A convenience sampling method was used for participant recruitment.

Statistical analysis will be conducted using IBM SPSS Statistics for Windows, Version 27 (Released 2019; IBM Corp., Armonk, New York, United States), and categorical variables will be presented in count and percentage. Quantitative variables will be presented in mean, standard deviation, median, minimum, and maximum. Comparison of quantitative variables between study groups was conducted using the Mann-Whitney U test. The Spearman correlation test assessed the correlation between the number of DDIs and other continuous variables; any p-value <0.05 was considered significant.

## Results

We enrolled 40 patients diagnosed with advanced malignancies, which were primarily managed by the palliative care team; they had nearly equal distribution of male patients (52.5%) and female patients (47.5%). The mean age is 74 years (±14.5), ranging from 27 to 93 years. Most patients have multiple chronic illnesses, with diabetes (47.5%) and hypertension (45.0%) being the most common. Additionally, 57.5% have other chronic conditions. Gastrointestinal and hepatocellular carcinoma were the most common cancers, making up over half of the cases (30% and 27.5%, respectively). Gynecological cancers and metastatic sarcoma each accounted for 12.5%, followed by genitourinary cancers (7.5%) and head and neck cancer (5%). Patients generally have an average of two chronic illnesses (±1.1), highlighting a significant comorbid burden (Table 1).

**Table 1:** Demographic and Clinical Characteristics of the Included Patients (n = 40) Data are presented as mean ± standard deviation (SD) and minimum–maximum (Min, Max) for continuous variables, and as counts and percentages for categorical variables. Other diseases include asthma, hypothyroidism, and autoimmune diseases.

Variable	Item	Mean ±SD	Min, Max
Age (years)		74±14.5	27, 93
		Count	%
Gender	Female	19	47.50%
	Male	21	52.50%
Chronic illnesses	Diabetes	19	47.50%
	Hypertension	18	45.00%
	Ischemic heart disease	6	15.00%
	Chronic kidney disease	5	12.50%
	Chronic liver disease	6	15.00%
	Cerebrovascular stroke	4	10.00%
	Other diseases	23	57.50%
Diagnosis	Gastrointestinal cancers	12	30%
	Hepatocellular carcinoma	11	27.50%
	Gynecological cancers	5	12.50%
	Metastatic sarcoma	5	12.50%
	Genitourinary cancers	3	7.50%
	Head and neck cancer	2	5%
	Glioblastoma multiforme	1	2.50%
	Chronic leukocytic leukemia	1	2.50%

**Figure 1:**
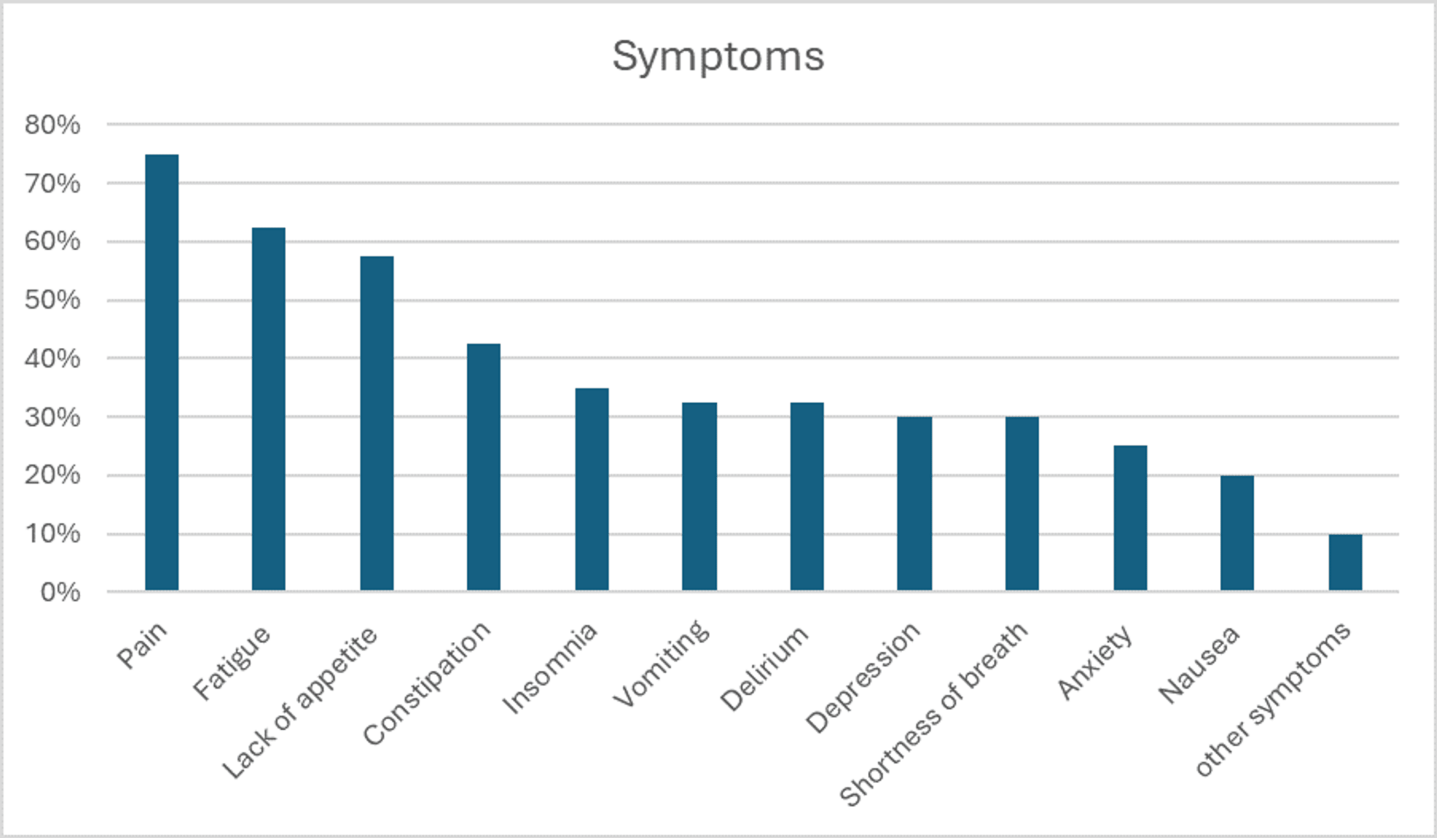
Chart Showing Symptoms Among the Included Patients Pain was the most presenting symptom, followed by fatigue, lack of appetite, constipation, and insomnia; least were anxiety and nausea.

Pain is the most prevalent symptom, affecting 75.0% of patients, with an average severity of 4.4 ± 3.5 on the ESAS scale (0-10). Fatigue is also highly reported (62.5%), followed by loss of appetite (57.5%) and constipation (42.5%). Psychological symptoms like anxiety (25.0%) and depression (30.0%) are also present but less severe (Table 2, Figure 1).

**Table 2:** Symptom Prevalence and Severity Scores Based on the Edmonton Symptom Assessment System (ESAS) (n = 40) This table presents the prevalence and severity of symptoms among patients, as measured by the Edmonton Symptom Assessment System (ESAS). Prevalence is reported as counts and percentages of patients who experienced each symptom. Symptom severity is reported using the ESAS, with values shown as mean ± standard deviation (SD) and median (minimum, maximum). The ESAS is a scale from 0 (no symptom) to 10 (worst possible severity).

Symptoms	Prevalence		ESAS Score	
	Count	%	Mean ±SD	Median (Min, Max)
Constipation	17	42.5%	1.8±2.7	0 (0, 9)
Vomiting	13	32.5%	1.2±2.2	0 (0, 9)
Pain	30	75.0%	4.4±3.5	5 (0, 10)
Delirium	13	32.5%	1.7±3.5	0 (0, 9)
Fatigue	25	62.5%	2.5±3	1.5 (0, 9)
Anxiety	10	25.0%	0.9±1.7	0 (0, 5)
Depression	12	30.0%	1±1.8	0 (0, 6)
Insomnia	14	35.0%	1.4±2.3	0 (0, 9)
Lack of appetite	23	57.5%	2.7±3.2	2 (0, 10)
Nausea	8	20.0%	0.8±2	0 (0, 10)
Shortness of breath	12	30.0%	1.3±2.4	0 (0, 8)
Other symptoms	4	10.0%	0.3±1	0 (0, 5)

**Figure 2:**
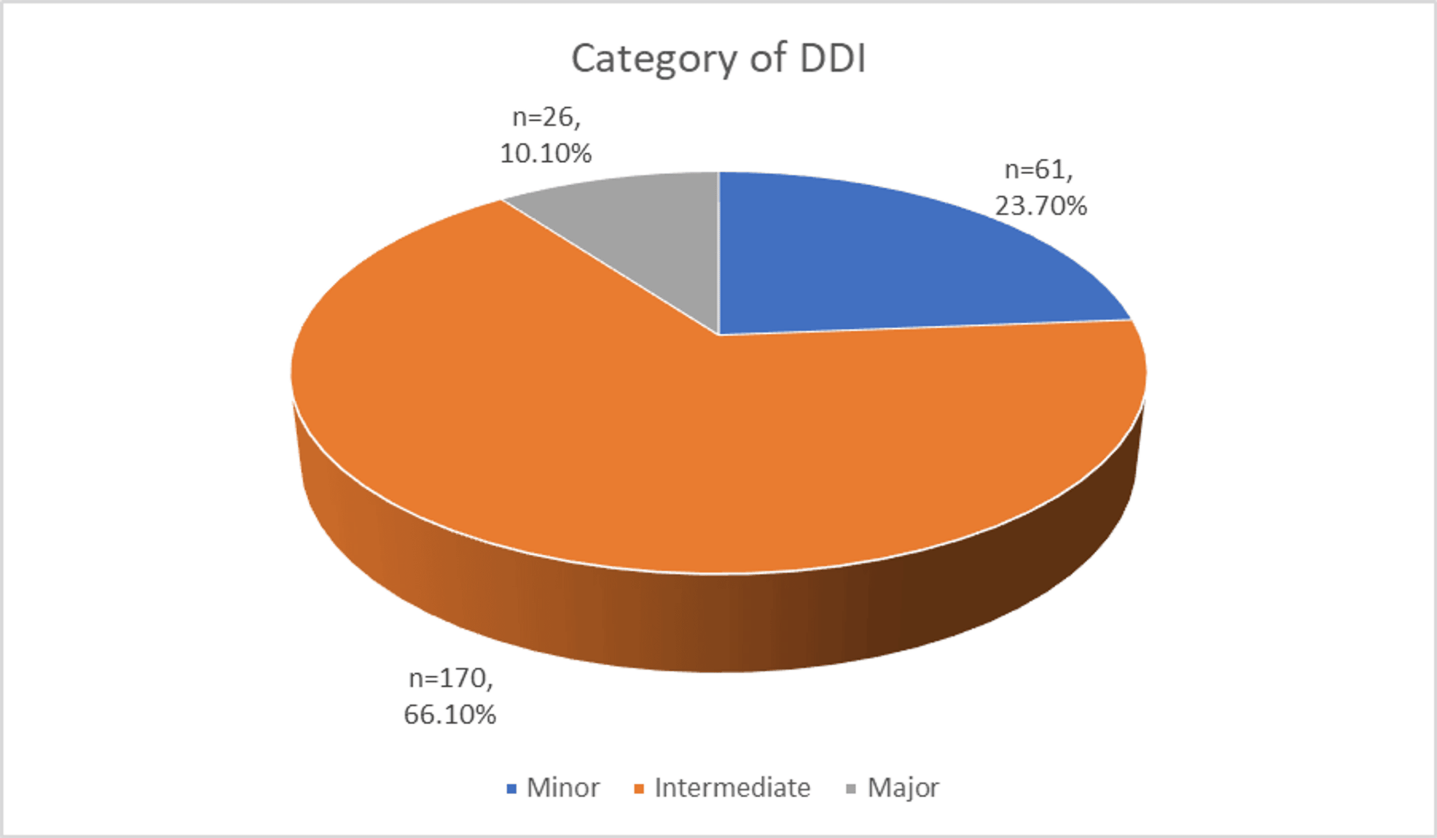
The Pie Chart Illustrating the Distribution of DDI Categories Among the Included Patients Intermediate interactions were the most common, followed by minor interactions, while major interactions were the least frequent DDI, drug-to-drug interaction

Seventy-five percent of patients experience at least one potential drug-to-drug interaction (pDDI), a total of 257 pDDIs. The majority are moderate (66.1%), followed by minor (23.7%) and major (10.1%) interactions (Figure 2). On average, each patient is on 10.3 ± 3.9 medications and experiences 6.4 ± 7.1 DDIs. Despite the high prevalence of pDDIs, only 17.5% of patients develop actual DDIs (aDDIs), with sedation (5.0%) and confusion (2.5%) being the most common symptoms. Primary management typically involved dose adjustments (15.0%), while most cases (82.5%) did not require intervention (Table 3).

**Table 3:** Drug-to-Drug Interactions, Classification, and Outcomes Among Study Participants (n = 40) This table summarizes the frequency and outcomes of potential and actual drug-to-drug interactions among 40 patients. It presents the number and percentage of patients who experienced DDIs, those who developed adverse events, the symptoms attributed to DDIs, actions taken by the treating team, and the DDI outcome. "Not applicable" refers to patients who did not experience a DDI or require intervention. pDDIs, potential drug-to-drug interactions; aDDIs, actual drug-to-drug interactions.

Variable	Item	Count	%
Number of patients with pDDIs	No	10	25.0%
	Yes	30	75.0%
Number of patients who developed aDDIs	No	33	82.5%
	Yes	7	17.5%
aDDI	Confusion	1	2.5%
	Electrolytes disturbances	1	2.5%
	Sedation	2	5.0%
	Others	3	7.5%
	Not applicable	33	82.5%
Actions taken by the primary treating team	Dose adjustment	6	15.0%
	Other actions	1	2.5%
	Not applicable	33	82.5%
Outcome of DDI	Resolved	7	17.5%
	Not applicable	33	82.5%

The presence of pDDIs does not significantly differ by gender (p=0.855) or age (p=0.89). Patients with DDIs tend to have more chronic illnesses (2.1 vs. 1.7, p=0.272) and a greater number of medications (10.7 vs. 9.2, p=0.396), though these differences are not statistically significant. Symptom burden is higher in those with DDIs, with notable differences in pain (p=0.062), depression (p=0.062), and appetite loss (p=0.050), suggesting a potential association between higher symptom severity and drug interactions (Table 4).

**Table 4:** Comparison of Clinical and Symptom Characteristics Between Patients With and Without Drug-to-Drug Interactions This table compares demographic, clinical, and symptom-related variables between patients who experienced drug-to-drug interactions (DDIs) and those who did not. Data are presented as mean ± standard deviation (SD), along with minimum and maximum values for each group. The Mann-Whitney U test (MW) was used for comparison. SD, standard deviation; Min, minimum; Max, maximum.

Variable	Drug-to-Drug Interactions					
	No		Yes			
	Mean ±SD	Min, Max	Mean ±SD	Min, Max	MW	p-value
Age (years)	73.9±13.9	50, 93	74.1±15	27, 93	0.156	0.890
Number of chronic illnesses	1.7±1.1	1, 4	2.1±1.1	1, 4	1.175	0.272
Number of symptoms	3.4±2.5	0, 8	4.9±3.1	0, 11	1.357	0.187
Number of medications	9.2±2.8	6, 14	10.7±4.2	5, 21	0.881	0.396
Constipation	1±1.6	0, 5	2.1±3	0, 9	0.660	0.569
Vomiting	0.3±0.7	0, 2	1.5±2.5	0, 9	1.202	0.331
Pain	2.6±3.2	0, 8	5±3.5	0, 10	1.898	0.062
Delirium	0.4±1	0, 3	2.2±3.4	0, 9	1.258	0.301
Fatigue	1.4±0.8	0, 2	2.9±3.3	0, 9	0.290	0.794
Anxiety	0.4±0.8	0, 2	1±1.9	0, 5	0.576	0.678
Depression	0±0	0, 0	1.3±2	0, 6	2.315	0.062
Insomnia	0.5±1.1	0, 3	1.7±2.5	0, 9	1.304	0.272
Lack of appetite	0.9±1.2	0, 3	3.3±3.4	0, 10	2.039	0.050
Nausea	0.3±0.9	0, 3	1±2.3	0, 10	0.939	0.528
Shortness of breath	0.6±1.3	0, 4	1.6±2.6	0, 8	0.724	0.724
Other symptoms	0.1±0.3	0, 1	0.4±1.2	0, 5	0.090	0.963

**Figure 3:**
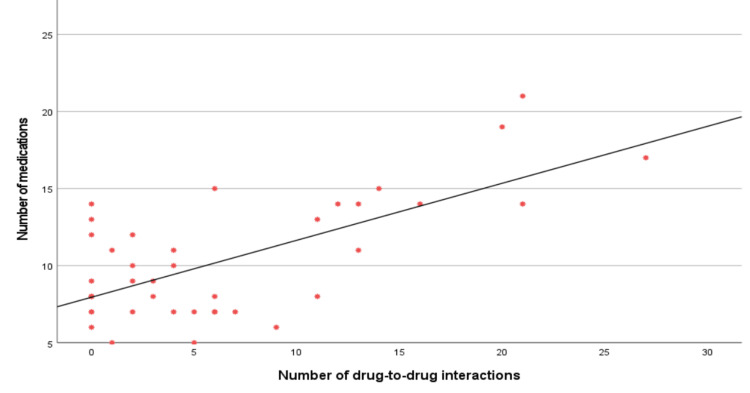
Scatter Plot Showing the Correlation between the Number of Medications and Number of pDDIs Among the Included Patients The number of medications was found to be directly correlated with the number of pDDIs pDDIs, potential drug-to-drug interactions

The number of medications correlates significantly with the number of pDDIs (r = 0.435, p = 0.005), reinforcing polypharmacy as a key risk factor. However, age, number of symptoms, and chronic illnesses do not show significant associations with DDIs (p>0.05) (Table 5, Figure 3).

**Table 5:** Spearman’s Correlation Between the Number of Potential Drug-to-Drug Interactions and Patient Characteristics This table shows the results of Spearman’s rank-order correlation (rho) between the number of drug-to-drug interactions (DDIs) and selected patient characteristics, including age, number of medications, symptoms, and chronic illnesses.

Variable	Potential Drug-to-Drug Interactions	
	Rho	p-value
Age (years)	0.011	0.945
Number of medications	0.435	0.005
Number of symptoms	0.114	0.484
Number of chronic illnesses	0.271	0.091

## Discussion

pDDIs represent a significant clinical challenge across healthcare settings, necessitating rigorous pharmacological surveillance, although elderly palliative care patients are a vulnerable group who usually exhibit polypharmacy and a variety of chronic illnesses. However, in the current study, we found that 75% of screened palliative patients had pDDIs; only 17.5% developed actual DDIs, most commonly confusion and sedation. The majority of DDIs were intermediate and were mainly correlated with the number of medications prescribed and symptoms such as pain, depression, and loss of appetite. This distinction between potential and actual DDIs is crucial and less commonly addressed in the literature.

To our knowledge, this is the first prospective study conducted in GCC countries that assesses the prevalence, severity, and actual incidence of DDIs among palliative care patients who are diagnosed with advanced malignancies.

Our findings were consistent with several retrospective studies across Europe, Canada, Australia, and the Middle East. The highest DDI prevalence was reported in an Iranian study, which highlighted that 91.7% of patients experienced at least one potential DDI during end-of-life care, 64.5% of which were intermediate and major DDIs [19]. Gaertner et al. found that 75% of palliative patients had at least one DDI, with 631 potential drug interactions identified [9]. Nonetheless, an Australian study analyzed palliative patients during the final two weeks of life; they found that 72% of patients were found to be at risk of at least one potential drug interaction, with only 2.4% patients developing actual symptoms of DDIs [20]. Additionally, in a retrospective study of 100 hospitalized cancer patients, 63% were found to have been exposed to at least one potentially interacting drug combination [7].

Conversely, many studies have reported much lower prevalence of DDIs compared to our study. A retrospective study performed in Toronto, Canada, found that among 372 palliative patients, 250 potential drug interactions were found in 115 patients (31%); the most frequently involved drugs were warfarin and phenytoin. Most interactions were categorized as moderately severe (59%) [6]. This discrepancy may be attributed to the differences in prescribing patterns, study design (retrospective), or patient selection criteria. Notably, our prospective approach allows for a more accurate estimation of clinically relevant DDIs, rather than theoretical potential interactions.

The literature consistently identifies polypharmacy as a primary risk factor for DDIs. Our findings reinforce this, highlighting the need to reconsider chronic illness management in the terminal phase. While managing comorbidities is important, our results and others suggest that a focus on symptom burden rather than disease-specific treatment may reduce unnecessary interactions and improve quality of life.

Consistent with our findings, studies reported that multivariable analysis showed that the risk of potential drug interactions increased with the number of medications prescribed (OR = 1.4 per additional drug, 95% CI = 1.26-1.58, p < .001). Additionally, patients receiving medications for comorbid conditions, rather than only supportive care drugs, had a significantly higher risk of drug interactions (OR = 8.6, 95% CI = 2.9-25, p < .001) [8].

The findings indicate that refining CDSS alerts, especially in palliative care, should consider the restricted relevance of DDI alerts for patients focused on symptom management and quality of life. Strict compliance with these alerts may not always align with patient-centered care objectives, emphasizing the importance of a more customized approach to CDSS use in palliative settings [20].

We recommend reducing the number of medications prescribed for managing chronic illnesses in the end-of-life stage. Instead, the focus should be on addressing the most distressing symptoms rather than treating each symptom individually. A holistic approach to symptom management is essential to improving patient comfort and quality of life during this stage. 

Future research should utilize multi-center longitudinal designs to further explore these clinical predictors. Implementing standardized medication reviews and pharmacist-led interventions may further enhance safety and patient-centered care at the end of life.

Limitations

This study is limited by its relatively small sample size and single-center design, which may affect the generalizability of the findings. Furthermore, although our prospective design improves the detection of actual adverse events, some DDIs may have gone unrecognized due to subtle or overlapping symptoms. The lack of a control group and detailed pharmacokinetic analysis also limits our ability to determine the causality of the observed actual DDIs.

## Conclusions

DDIs are relatively common among palliative care patients, but clinically significant adverse events are infrequent. Given the small sample size and single-center design, findings should be interpreted with caution. Regular medication reviews and tailored prescribing may support safer, more patient-centered care at the end of life.
